# 

**Published:** 1899-04

**Authors:** 


Vol. LIII. APRIL, 1899. No. 4.

LIBRARY
Indexed
S. G. O.

### THE
### DENTAL REGISTER

A MONTHLY JOURNAL.

Devoted to the Interests of the Profession.

EDITED BY

J. TAFT, D. D. S.

LIBRARY

SURGEON GENERAL'S OFFICE

APR.-24.-1899

PUBLISHED BY
SAM'L A. CROCKER & CO.,
OHIO DENTAL AND SURGICAL DEPOT,
35, 37 and 39 West Fifth Street, CINCINNATI, OHIO.

Entered at the Postoffice at Cincinnati, O., as second class mail matter.

TERMS: $2.00 per Annum in Advance. Single Copies, 25 Cts.

